# Supplementary material for: Association of Maternal Antenatal Education with Quality of Life after Childbirth in the Slovenian Population before and during the COVID-19 Pandemic
Source: Healthcare (Basel). 2023 May 26;11(11):1568. doi: 10.3390/healthcare11111568 (PMC10252583; doi:10.3390/healthcare11111568)
Supplement: Supplementary file 1 [file healthcare-11-01568-s001.zip › healthcare-2397970-supplementary.pdf]

Table S1: POST PARTUM QUALITY OF LIFE QUESTIONAIRE

Items	Item content	1	2	3	4	5
PQOL1	Do you worry that your child will fall sick?					
PQOL2	How satisfied are you with your child's health?					
PQOL3	Do you worry that your child will have an accident?					
PQOL4	How much do you take pains to prevent an accident to your child?					
PQOL5	Do you worry about the nutrition of your child?					
PQOL6	Do you worry that your child is not smart?					
PQOL7	Do you think that your breast milk is enough for your child?					
PQOL8	How satisfied are you with current feeding?					
PQOL9	Do you worry about unexpected pregnancy?					
PQOL10	How much are you bothered by contraception?					
PQOL11	How satisfied are you with your sleep?					
PQOL12	Do you have enough time to rest?					
PQOL13	How easily do you get tired?					
PQOL14	How satisfied are you with the energy that you have?					
PQOL15	Does physical pain influence your daily life?					
PQOL16	How much do you think that your physical health has been affected by childbirth?					
PQOL17	How much conflict do you feel between child care and work?					
PQOL18	Has your child caused you to be distracted and worried at work?					
PQOL19	How satisfied are you with the way your body looks?					
PQOL20	Do you feel blue by your looks?					
PQOL21	How much confidence do you have in caring for your baby well?					
PQOL22	How much child care skill do you think you have?					
PQOL23	Are you interested in your child?					
PQOL24	Are you willing to look after your child?					
PQOL25	Do you regret having had this child?					
PQOL26	Is caring a baby hard for you?					
PQOL27	Are you happy being a mother?					
PQOL28	How much fun is your life after having this child?					
PQOL29	Do you have enough contact with the outside world?					
PQOL30	Do you see enough of your neighbors?					
PQOL31	What do you think your husband's attitude is towards you?					
PQOL32	How close is the relationship between you and your husband?					
PQOL33	How much help do you get caring for your child?					
PQOL34	How much help do you get doing household chores?					
PQOL35	How clean is your house?					
PQOL36	How satisfied are you with your housing situation					
PQOL37	Is the money that yourself can decide how to spend enough?					
PQOL38	Do you worry about your finances?					
PQOL39	How satisfied are you with your living environment, including pollution, noise, climate, and location?					
PQOL40	How satisfied are you with the transportation available to you?					

Abbreviation: PQOL: Postpartum Quality of Life
For items 1, 3, 4, 5, 6, 9, 12, 15, 23, 24, 25, 26, 27, 38, the responses were; 1: Never, 2: Rarely, 3: Sometimes, 4: Often and 5: Always.
For items 2, 8, 11, 14, 19, 36, 39, 40, the responses were; 1: Very Unsatisfied, 2: Unsatisfied, 3: Neither satisfied nor unsatisfied, 4: Satisfied, 5: Verysatisfied.
For items 7, 37, the responses were; 1: Is not enough at all, 2: Is not enough, 3: Sometimes, 4: Is enough, 5: Is always enough.
For items 10, 13, 16, 17, 18, 20, 21, 22, 28, 29, 30, 32, 33, 34, 35, the responses were; 1: Not at all, 2: Little, 3: Moderately, 4: Very, 5: Too much.And for item, 31 the responses were; 1: Very bad, 2: Bad, 3: Neither bad nor good, 4: Good, 5: Very good.
